# Genome Sequences of 13 Isolates of Salmonella enterica Serovar Typhimurium var. Copenhagen Obtained from Wild Pigeons in Canada

**DOI:** 10.1128/genomeA.00392-18

**Published:** 2018-05-17

**Authors:** Bridget Xie, Andrée Ann Dupras, Marc-Olivier Duceppe, Nooshin Fattahi-Ghazi, Lawrence Goodridge, Dele Ogunremi

**Affiliations:** a Department of Food Science and Agricultural Chemistry, Faculty of Agricultural and Environmental Sciences, Macdonald Campus, McGill University, Sainte-Anne-de-Bellevue, Quebec, Canada; b Canadian Food Inspection Agency, Ottawa Laboratory Fallowfield, Ottawa, Ontario, Canada

## Abstract

Pigeon-adapted strains of Salmonella enterica serovar Typhimurium var. Copenhagen phage types 2 and 99 obtained from the provinces of Alberta, British Columbia, and Ontario, Canada, were analyzed using whole-genome sequencing. All isolates contained the Salmonella virulence plasmid despite the low pathogenicity of this lineage in their avian host.

## GENOME ANNOUNCEMENT

The prevalence of foodborne salmonellosis in North America has remained high despite improved diagnostic tools and many years of investments in food testing. Efforts to disrupt the dissemination of Salmonella spp. from their sources into the food chain have met with limited success, and often, the source of contamination is unknown. Cases of foodborne salmonellosis of bird origin have been well documented in the literature (1–3), but a quantitative assessment of risk posed by birds in the spread of foodborne salmonellosis in Canada or elsewhere is lacking. Pigeons carry host-adapted strains of Salmonella enterica serovar Typhimurium var. Copenhagen phage type 2 (PT2) and PT99, often without showing clinical signs (1, 4). Consequently, these host-adapted Salmonella strains are considered an insignificant public health risk (4). However, Salmonella enterica serovar Typhimurium PT2 has been isolated from human clinical cases in Canada (5), and experimental infection with PT99 can result in death in pigeons (6). A previous evaluation of a single genome of Salmonella Typhimurium PT99 did not reveal any distinguishing features from other PTs infecting other species (7). Thus, host adaptation may well be the consequence of complex interactions between the organism and its host and could be overwhelmed by exposure to large doses (6). Genome analysis of multiple isolates of this Salmonella lineage should help identify genetic attributes that contribute to host adaptations and virulence factors that could help evaluate the potential risk to food safety if introduced into the food chain. To that end, we have sequenced the genomes of 13 isolates of Salmonella Typhimurium from dead pigeons submitted as part of wild bird surveillance programs in the provinces of Alberta, British Columbia, and Ontario, Canada. Nine isolates belonged to PT2, while the remaining four were designated PT46, PT99, PT193, and atypical (Table 1).

**TABLE 1:** Salmonella enterica serovar Typhimurium var. Copenhagen of pigeon origin

Isolate	Province^*a*^	Phage type	Accession no.
OLF-FSR1-ST-44	BC	2	PYUR00000000
OLF-FSR1-STCopenhagen-46	ON	Atypical	PYUS00000000
OLF-FSR1-STCopenhagen-47	BC	2	PYUT00000000
OLF-FSR1-STCopenhagen-48	BC	2	PYUU00000000
OLF-FSR1-STCopenhagen-49	BC	193	PYUV00000000
OLF-FSR1-STCopenhagen-50	ON	2	PYUW00000000
OLF-FSR1-STCopenhagen-51	ON	2	PYUX00000000
OLF-FSR1-STCopenhagen-52	ON	2	PYUY00000000
OLF-FSR1-STCopenhagen-53	ON	2	PYUZ00000000
OLF-FSR1-STCopenhagen-55	BC	2	PYVA00000000
OLF-FSR1-STCopenhagen-56	BC	46	PYVB00000000
OLF-FSR1-STCopenhagen-57	ON	2	PYVC00000000
OLF-FSR1-STCopenhagen-SA20132913	AB	99	PYVD00000000

a BC, British Columbia; ON, Ontario; AB, Alberta.

We performed Illumina MiSeq whole-genome sequencing on DNA libraries constructed with a TruSeq kit and used the version 3 sequencing kit, according to the manufacturer’s instructions (Illumina, San Diego, CA). Quality trimming and filtering of Illumina reads were performed using the BBTools software suite (http://jgi.doe.gov/data-and-tools/bbtools/). Assembly of Illumina paired-end reads was performed with SPAdes version 3.11.1 (8) and polished with Pilon version 1.22 (9) using Unicycler version 0.4.4 (10) (https://github.com/rrwick/Unicycler). Annotation of the final assemblies was done using the National Center for Biotechnology Information Prokaryotic Genome Annotation Pipeline (11), and the presence of antimicrobial resistance (AMR) genes was investigated with ResFinder (12). The estimated mean genome size and standard deviation were 4,859,860 ± 17,288 bp, and the genomes contained an average of 4,580 ± 25 coding sequences and 72 ± 1 transfer RNAs. The large virulence plasmid of Salmonella spp. was demonstrated in all isolates, as confirmed by reference assembly with a published plasmid sequence (strain 22495, accession number CP017618 [13]). AMR genes were not found in any of the isolates using ResFinder. These genomes should allow for a detailed comparison of the attributes of Salmonella Typhimurium isolates from pigeons with those of other wild birds and with isolates contaminating the food chain.

### Accession number(s).

The nucleotide sequences for the chromosome and plasmids have been deposited at DDBJ/ENA/GenBank under BioProject number PRJNA434296. GenBank accession numbers are listed in Table 1.
